# On the Generation and Regeneration of Retinal Ganglion Cells

**DOI:** 10.3389/fcell.2020.581136

**Published:** 2020-09-17

**Authors:** Viviane M. Oliveira-Valença, Alejandra Bosco, Monica L. Vetter, Mariana S. Silveira

**Affiliations:** ^1^Laboratory of Neurogenesis, Neurobiology Program, Institute of Biophysics Carlos Chagas Filho, Federal University of Rio de Janeiro, Rio de Janeiro, Brazil; ^2^Department of Neurobiology and Anatomy, University of Utah, Salt Lake City, UT, United States; ^3^Glial Cell Biology Group, Instituto de Investigação e Inovação em Saúde, Universidade do Porto, Porto, Portugal

**Keywords:** regeneration, retinal ganglion cells, glaucoma, development, molecular programs

## Abstract

Retinal development follows a conserved neurogenic program in vertebrates to orchestrate the generation of specific cell types from multipotent progenitors in sequential but overlapping waves. In this program, retinal ganglion cells (RGCs) are the first cell type generated. RGCs are the final output neurons of the retina and are essential for vision and circadian rhythm. Key molecular steps have been defined in multiple vertebrate species to regulate competence, specification, and terminal differentiation of this cell type. This involves neuronal-specific transcription factor networks, regulators of chromatin dynamics and miRNAs. In mammals, RGCs and their optic nerve axons undergo neurodegeneration and loss in glaucoma and other optic neuropathies, resulting in irreversible vision loss. The incapacity of RGCs and axons to regenerate reinforces the need for the design of efficient RGC replacement strategies. Here we describe the essential molecular pathways for the differentiation of RGCs in vertebrates, as well as experimental manipulations that extend the competence window for generation of this early cell type from late progenitors. We discuss recent advances in regeneration of retinal neurons *in vivo* in both mouse and zebrafish and discuss possible strategies and barriers to achieving RGC regeneration as a therapeutic approach for vision restoration in blinding diseases such as glaucoma.

## Introduction

RGCs are the output neurons of the retina, connecting to brain targets through the optic nerves. Recent single-cell RNA-seq (scRNAseq) studies in the mouse retina have identified 46 transcriptional RGC subtypes (Laboissonniere et al., 2019; Tran et al., 2019). Clusters of RGC subtypes are also defined by properties such as the response to light stimulation, preference for local motion, uniform illumination or motion direction, dendritic morphology and lamination (Kong et al., 2005; Coombs et al., 2006; Laboissonniere et al., 2019; Tran et al., 2019). Although a great variety of visual attributes are codified by these RGC subtypes, the molecular mechanisms responsible for generating this diversity are not completely understood.

In vertebrates it is well established that RGCs are among the earliest-born cell types. In chicken, RGC generation starts at embryonic day 2, E2 (Prada et al., 1991; Zhang et al., 2018), in zebrafish 27–28 hpf (hours post fertilization) (Hu and Easter, 1999), in *Xenopus*, between stages 24 and 29 (Holt et al., 1988), and in the mouse, from E11 up to postnatal day 0 (P0, corresponding to around E19), with a peak at E14 (Drager, 1985; Young, 1985). In human embryonic retina RGC neurogenesis starts at the 7^th^ gestation week, and transcriptomic and scRNAseq analysis showed similarity in cell specification timing as compared to mice (Aldiri et al., 2017; Hoshino et al., 2017; Lu et al., 2020).

**FIGURE 1:**
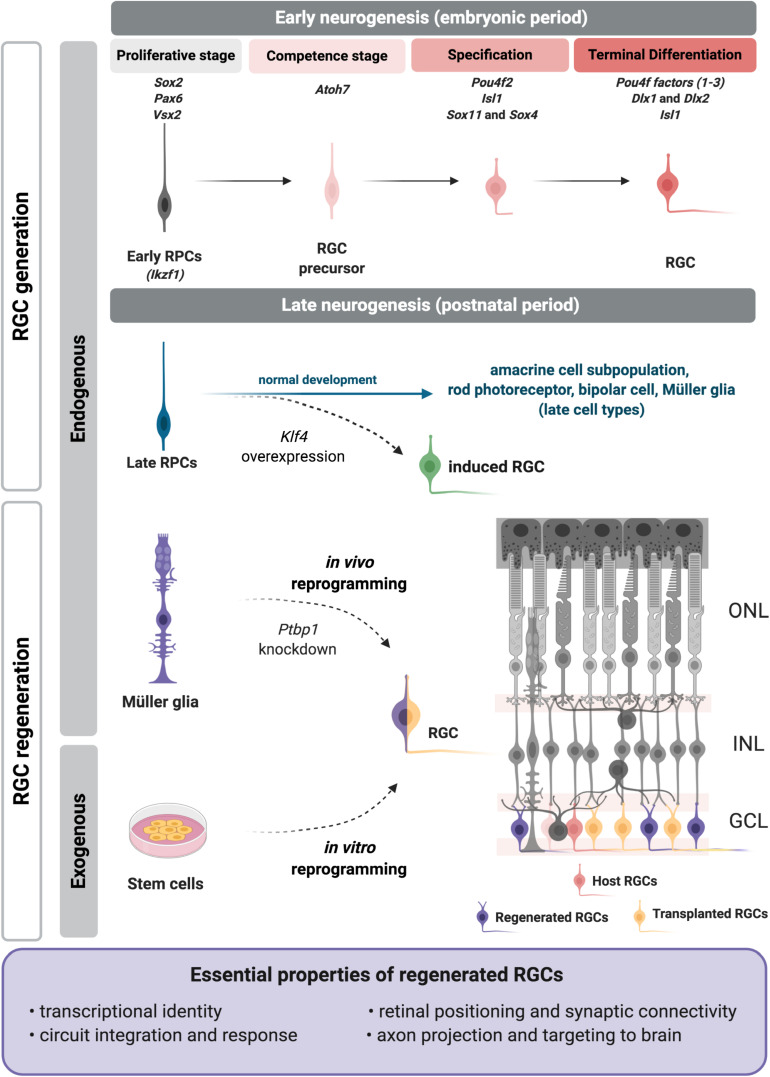
Regenerative approaches for retinal ganglion cell (RGC) replacement. During retinal development RGCs are generated from embryonic progenitors through a network of transcription factors. The critical factors are included above each developmental step at the top of the figure. Recently we showed that a RGC program may be reactivated in late RPCs upon *Klf4* overexpression (Rocha-Martins et al., 2019) to generate induced RGCs (green). Current RGC regenerative approaches apply strategies to induce or reactivate the embryonic molecular program on exogenous (induced pluripotent or embryonic stem cells) or endogenous (Müller glia) sources (left). Transplanted (yellow) or induced RGCs (purple) must meet essential properties (frame), as they integrate in the retina, such as the host RGCs (pink). RPCs, retinal progenitor cells; ONL, outer nuclear layer; INL, inner nuclear layer; GCL, ganglion cell layer. Figure created with BioRender.com.

Although much remains to be learned regarding the mechanisms underlying RGC generation, a hierarchical organization of transcription factors (TFs) has been defined that constitute a gene regulatory network in early progenitors essential to determine RGC competence, specification and terminal differentiation through the expression of critical effector genes (Figure 1; Boije et al., 2014; Mellough et al., 2019; Nguyen-Ba-Charvet and Rebsam, 2020). While most of this has been studied in model organisms, particularly mouse and zebrafish, relevant information has recently been generated from the study of retinal organoids, which allows the characterization of the molecular programs for the generation and diversification of cell types (Hoshino et al., 2017; Fligor et al., 2018), as well as comparison with developing human retina (Hoshino et al., 2017; Lu et al., 2020; Sridhar et al., 2020). ScRNA-seq coupled with pseudotime analysis of human retinal organoids or fetal retina have identified developmental trajectories from RPCs to each major cell type, including RGCs. This showed conservation of key regulators of RGC differentiation, as well as human-specific expression of MYC (Lu et al., 2020). It is striking that the conservation of developmental molecular programs between species is high. It will be interesting to characterize which information might be essential for the functionality of specific cell types in human retina.

## Molecular Program for RGC Generation

### Temporal Patterning of Retinal Progenitors

Across vertebrate species, the temporal sequence of cell genesis for the seven major classes of retinal cell types is evolutionarily conserved, with RGCs as the first cell type generated (Young, 1985; Turner et al., 1990; Cepko et al., 1996; Rapaport et al., 2004). Retinal cells are generated in sequential but overlapping waves from multipotent retinal progenitor cells (RPCs) that change their capacity to generate specific cell types, according to the “competence model” (Cepko et al., 1996). However, the mechanisms underlying this temporal control are not well understood.

There is evidence for intrinsic changes in competence states of RPCs over time (Cepko, 2014). For example, aggregates of RPCs cultured *in vitro* recapitulate the composition of clones *in vivo* (Gomes et al., 2011), and RPCs maintain their potency when transplanted to an earlier or older environment (Watanabe and Raff, 1990; Belliveau and Cepko, 1999; Belliveau et al., 2000). A temporal patterning of early and late RPC populations has been distinguished by single cell analysis of developing mouse retina (Clark et al., 2019), and the developing human retina (Lu et al., 2020). Some authors have proposed that the fate of RPCs could be partially stochastic (Gomes et al., 2011; He et al., 2012). Also, extrinsic signals can influence the timing and competence of cell type generation, including RGCs (reviewed by Mills and Goldman, 2017). For example, there is a gradient of increasing Notch pathway gene expression in progenitors as development progresses (Clark et al., 2019). Feedback mechanisms, such as Shh and GDF11 for RGCs, can also limit the number of a given cell type produced (Kim et al., 2005; Wang et al., 2005).

One of the first studies to propose molecular mechanisms for the temporal control of cell identity acquisition described the roles of specific transcription factors in Drosophila, with *hunchback* (*Hb*) regulating the transition from early to late progenitors (Isshiki et al., 2001). Its mouse ortholog, *Ikaros* (*Ikzf1*) has the same role in early RPCs, and its loss of function leads to fewer early-born cell types such as RGCs, but does not affect late-born cell types (Elliott et al., 2008). *Casz1*, another ortholog of fly transcription factor- *castor*-, regulates the fate of mid/late born cell types and suppresses the generation of early-born cell types, as shown by conditional deletion (Mattar et al., 2015). Furthermore, *Casz1* is repressed by *Ikaros* (*Ikzf1*), as shown in Drosophila for *castor* and *hunchback* (Mattar et al., 2015). The potential roles of other elements of this network, like fly *Krüppel* and *Pdm*, remain unknown. Recently, *Klf4*, a member of the family of *Krüppel*-like factors was studied in the mouse retina, but no critical function in cell fate determination was described (Moore et al., 2009; Fang et al., 2016; Rocha-Martins et al., 2019). This may be due to redundancy with other Klf family members (Jiang et al., 2008), since many are expressed in the developing retina (Moore et al., 2009; Njaine et al., 2014). We recently showed that overexpression of *Klf4* in late retinal progenitors generates induced RGCs outside of their developmental window (Figure 1; Rocha-Martins et al., 2019). This study showed that *Klf4* induced the reactivation of the early neurogenic program in late progenitors, changing their competence to generate RGCs that properly localized to the inner retina and projected axons into the optic nerve head (Rocha-Martins et al., 2019). The precise mechanism underlying the effect of *Klf4* in late progenitors is still unknown, but we hypothesize that *Klf4* reactivates the molecular program for RGC differentiation through its properties as a pioneer factor, combined with the direct or indirect induction of *Atoh7* (Chronis et al., 2017; Rocha-Martins et al., 2019). Although these results are promising, the detailed characterization of the transcriptional signature, subtype, and function of these induced RGCs, as well as their capacity to connect within the retina and with their brain targets remains to be defined. It will be intriguing to determine whether *Klf4* could also be used to promote or enhance the reprogramming of postmitotic retinal cells to generate induced RGCs for regeneration.

### miRNA and Epigenetic Regulation of Progenitor Competence

miRNAs also play a role in the control of the transition of competence from early to late progenitors (Decembrini et al., 2009; Georgi and Reh, 2010; Davis et al., 2011). Retinal-specific deletion of *Dicer* results in prolonged production of RGCs beyond the normal competence window and failure to produce later-born cell types (Georgi and Reh, 2010). Three miRNAs, let-7, miR-125, and miR-9 are critical regulators of this early to late competence transition, and their overexpression can rescue the progression to late progenitors in Dicer-cKO (conditional knockout) (La Torre et al., 2013). *Lin28* and *Prtg* are targets of these miRNAs and can maintain the early progenitor state when overexpressed, however overexpression in late progenitors was not sufficient for them to reacquire the early progenitor state since only very rare Brn3+ cells were observed in the neuroblastic layer (La Torre et al., 2013).

Besides transcription factor networks, the control of chromatin landscapes is relevant for the establishment of the competence transitions throughout retinal development (Aldiri et al., 2017; Zibetti et al., 2019). For example, in both human and mouse retina changes in histone modifications, particularly repressive H3k27me3, are associated with developmental transitions in the expression of differentiation programs for specific cell types (Aldiri et al., 2017). In addition, conditional disruption of the repressive histone H3K27 trimethylase *Ezh2* in RPCs results in accelerated onset of differentiation for late-born retinal cell types (Iida et al., 2015; Zhang et al., 2015). How regulation of chromatin contributes to differentiation of early cell types, including RGCs, remains to be elucidated.

### Transcriptional Regulation of RGC Development

Early in retinal development, transcription factors such as *Sox2*, *Pax6*, and *Vsx2*/*Chx10* regulate the proliferation of multipotent retinal progenitors as well as the expression of critical competence factors. While *Pax6* induces *Atoh7/Math5* expression in early development (Riesenberg et al., 2009), *Vsx2* represses the expression of this transcription factor (Burmeister et al., 1996; Marquardt et al., 2001; Vitorino et al., 2009). The disruption of this repression is critical as *Atoh7* is necessary to confer competence to RPCs to generate RGCs. Although *Atoh7* is not sufficient for RGC differentiation and is expressed in progenitors that generate a range of cell types (Brown et al., 2001; Lu et al., 2020), its absence leads to the loss of about 80% of RGCs in mice (Brown et al., 2001; Wang et al., 2001) and of almost all RGCs in zebrafish (Kay et al., 2001). Interestingly, *Atoh7* expression is transitory (Kanekar et al., 1997; Brown et al., 1998; Kay et al., 2001; Liu et al., 2001) and is regulated by itself as well as many transcription factors, such as *Pitf1a, Ngn2*, and *Neurod4*/*NeuroM*/*Atoh3* (Fujitani et al., 2006; Hernandez et al., 2007). At least one of these factors, *Pitf1a*, is directly regulated by *Foxn4* (Fujitani et al., 2006), which was recently shown to control RPC temporal identities and to suppress the RGC fate (Liu et al., 2020).

Downstream of *Atoh7* a plethora of transcription factors are essential for the generation, survival, and maturation of RGCs. *Atoh7* directly regulates *Pou4f2/Brn3b* expression and acts upstream of the other POU domain factors, *Pou4f1/Brn3a* and *Pou4f3/Brn3c* (Liu et al., 2001; Pan et al., 2005). These transcription factors are essential for terminal differentiation, survival and axonogenesis in RGCs, but not for initial fate specification (Wang et al., 2002; Pan et al., 2005; Badea et al., 2009). In addition, *Pou4f2* represses genes responsible for the differentiation program of other cell types (Qiu et al., 2008). *Atoh7* also regulates expression of *Isl1*, which acts in parallel but also in coincident subpopulations of RGCs with *Pou4f2* (Mu et al., 2008; Pan et al., 2008). These two factors work together to specify and differentiate the RGCs (Pan et al., 2008; Li et al., 2014; Wu et al., 2015). Analysis of *Atoh7*-expressing retinal progenitors revealed EYA2 as a protein phosphatase upstream of *Pou4f2* and involved in RGC specification (Gao et al., 2014).

The distal-less homeobox family of transcription factors, namely *Dlx1 and Dlx2*, are also relevant for both RGC survival and terminal differentiation (de Melo et al., 2003; Zhang et al., 2017). Their expression is regulated by *Atoh7* and they are direct regulators of *Pou4f2* expression, although they can also act in parallel with this transcription factor, as suggested by the study of triple knockout mice (Zhang et al., 2017). SoxC transcription factors are also important for RGC specification, with known roles for *Sox11* and *Sox4*. *Sox11* is expressed in early progenitors (Usui et al., 2013) and its loss delays RGC neurogenesis, although *Sox4* may compensate for *Sox11* since just a small reduction in RGC number was detected in late development (Jiang et al., 2013). The combined depletion or overexpression of *Sox11* and *Sox4* has shown that these transcription factors are not only necessary but sufficient for RGC differentiation, with their loss resulting in complete absence of the optic nerve (Chang et al., 2017). In addition, *Sox4*-dependent posttranslational modification of *Sox11* regulates its nuclear localization and activity. SoxC factors act upstream of Pou4f/Brn3 factors, although it is not known if they regulate them directly (Chang et al., 2017). Thus, a complex network of genes ultimately regulates the genesis and differentiation of RGCs from RPCs.

## Knowledge From Retinal Development as Tools for RGC Regeneration

### Retinal Ganglion Cell Loss in Disease

Being the sole output neurons of the retina and incapable of axon regeneration, RGC loss due to injury or disease results in permanent vision reduction and blindness. Several conditions impact RGC function and viability, including traumatic optic injury, ischemic injury, demyelinating and hereditary optic neuropathies, and diabetic retinopathy (Newman, 2012; Biousse and Newman, 2015; Altmann and Schmidt, 2018). Additionally, RGCs are the primary target of glaucoma, a group of neurodegenerative diseases characterized by progressive optic nerve axon damage and RGC death (Quigley, 2011; Calkins, 2012). Current treatments effectively control concomitant ocular hypertension, but not the progression of RGC neurodegeneration (Calkins, 2012; Fry et al., 2018). At present, there is no restorative treatment for reduced or lost vision due to loss of RGCs. This reinforces the relevance of investigating new therapeutic approaches.

### Regeneration From Endogenous Sources: Müller Glia

There are some lines of investigation aimed at developing innovative regenerative strategies based on RGC replacement. One of them invests in the transplantation of cells differentiated *in vitro* from stem cells (reviewed in Miltner and La Torre, 2019 and not covered here). The other aims to generate these cells from endogenous sources, redeploying the regenerative capacity present in teleost, but lost in mammals (Goldman, 2014). In both scenarios, researchers apply knowledge of fundamental processes for RGC development to design tools to open or expand a window for the generation of new or induced RGCs capable of surviving and making correct synaptic connections to restore visual function.

When considering new approaches to generate induced RGCs *in vitro* or *in situ*, a promising candidate is Müller glia, a well-defined endogenous source for retina regeneration (Goldman, 2014; Vetter and Hitchcock, 2017; Lahne et al., 2020). Müller glia are generated in the second wave of retinogenesis (Cepko et al., 1996) and are transcriptionally similar to late retinal progenitors (Blackshaw et al., 2004; Ooto et al., 2004; Jadhav et al., 2009; Nelson et al., 2011). In teleost fish Müller glia respond to injury, dedifferentiate to a progenitor-like profile, proliferate, generate all cell types and restore vision (Goldman, 2014). However, this regenerative potential has been lost (or actively suppressed) during evolution, and mammalian Müller glia possess reduced proliferative or neurogenic potential (Dyer and Cepko, 2000; Karl et al., 2008; Hamon et al., 2019; Rueda et al., 2019). Müller glia in chick have intermediate regenerative potential and retain the ability to dedifferentiate and adopt a proliferative progenitor-like state during a narrow window after hatching (Fischer and Reh, 2001). Thus, recent efforts are focused on comparative approaches to define injury-induced changes in Müller glia that may account for differences in reprogramming potential across species (Lahne et al., 2020).

In zebrafish retina, many signaling pathways are important for generation of Müller glia-derived progenitors, proliferation, and neurogenic potential, either in damage or disease contexts. For example, Wnt/ß-catenin is upregulated in response to damage and is critical to stimulate Müller glia proliferation (Ramachandran et al., 2011; Meyers et al., 2012; Yao et al., 2016). Notch and Fgf8a-to-Notch signaling are important regulators of Müller glia proliferation in zebrafish, and different outcomes distinguishes multiple populations of Müller glia (Wan and Goldman, 2017). EGF is secreted by Müller glia upon damage and induces its proliferation even when damage is absent (Wan et al., 2012). Interestingly, it was recently suggested that Hippo/YAP signaling may actively repress the proliferation of Müller glia in mice, and overexpression of a YAP form insensitive to phosphorylation is sufficient to induce Müller cell reprogramming into a highly proliferative cell (Hamon et al., 2019; Rueda et al., 2019). Moreover, activation of TGFß by metalloproteinases can influence Müller glia reprogramming and retina regeneration in zebrafish through multiple targets (Sharma et al., 2020). How all these signaling pathways are integrated is still under debate (Wan and Goldman, 2016; Lahne et al., 2020).

The search for ways to unlock this regenerative potential in mammals has increasingly attracted interest (Karl et al., 2008; Loffler et al., 2015; Ueki et al., 2015; Jorstad et al., 2017; Guimaraes et al., 2018; Yao et al., 2018). Pollak et al. (2013) demonstrated that the overexpression of *Ascl1* in Müller glia cultures and mouse retinal explants change gene expression with downregulation of glial genes and upregulation of progenitor genes. Moreover, they demonstrated *in vivo* the generation of cells with neuronal properties of amacrine, bipolar or photoreceptor cells. The use of an HDAC inhibitor to interfere with chromatin accessibility was effective also in mature retinas (Pollak et al., 2013; Ueki et al., 2015; Jorstad et al., 2017). Recently, the combination of *Ascl1* overexpression with the use of a STAT inhibitor in addition to the HDAC inhibitor, showed increased efficiency in generating bipolar neurons (Jorstad et al., 2020). This is promising, because confirms that it is possible to reactivate a program for neuronal generation in mammalian Müller glia. It is likely that regenerative approaches will have to be designed for specific cell types and disease contexts, particularly for regeneration of RGCs.

### Directed Strategies for Inducing Retinal Ganglion Cells

Strategies to reprogram existing cells to generate RGCs have been limited in mammalian retina, highlighting the need for innovative approaches. A preprint from Xiao et al. (2019) have described the generation of induced RGCs from Müller glia in mice through the overexpression of *Atoh7* and *Pou4f2*/*Brn3b*. These cells projected axons to superior targets in the brain and restored the vision in a disease model. In addition to Müller glia, an alternative endogenous source for the generation of induced RGCs could be another retinal neuron (Vetter and Hitchcock, 2017). Interestingly, Chen et al. (2015) proposed that a subpopulation of amacrine cells had regenerative potential.

Since the epigenetic landscape is important not only for proper tissue development, but also for cell reprogramming, strategies that target chromatin remodeling could also prove fruitful for promoting RGC generation. During Müller glial cell reprogramming in zebrafish changes in DNA methylation as well as histone modification are tightly regulated to promote both activation and repression of gene expression, although the role of epigenetic changes in regulating this process remains to be more fully defined (Lahne et al., 2020). Notably, the transcriptional repressor REST broadly represses neuronal gene expression in non-neuronal cells and in progenitors via recruitment of histone deacetylases (Lunyak et al., 2004). Many *Atoh7*-dependent genes, including *Pou4f2*, have REST-dependent repressor element 1 (RE1) sites (Mu et al., 2005). Release of REST-mediated repression plays an important role in activating RGC genes in RPCs, and in retinas with conditional deletion of *REST* the numbers of RGCs increased significantly (Mao et al., 2011). Thus, it is possible that relieving epigenetic constraints on RGC gene expression may enhance the generation of RGCs outside the normal developmental window. Consistent with this, it was recently shown that CRISPR-CasRx-mediated down regulation of the RNA-binding protein, *Ptbp1*, converts Müller glia to RGCs in mature retina *in vivo*, with projection of axons to brain and restoration of visual responses (Zhou et al., 2020). Reduced expression of *Ptbp1* was previously shown to convert fibroblasts to neurons *in vitro* through regulation of a miRNA targeting of multiple components of REST (Xue et al., 2013).

## Conclusion and Future Directions

Much effort has been invested into neuroprotective approaches and the control of risk factors that contribute to the degeneration of RGCs, such as intraocular pressure (IOP) in glaucoma. However, to restore vision it is essential to unravel innovative therapeutic strategies to replace damaged or lost RGCs and their connection to the appropriate superior targets. Here we discussed the principles of RGC generation throughout retinal development and considered new paths for regeneration based on the reactivation of developmental programs in combination to other strategies, such as interference with chromatin accessibility. The final goal would be to identify effective tools to extend or reopen the temporal window for RGC generation and apply it to replacement approaches (Figure 1). Potential candidates to apply such approaches would be Müller glia or other retinal neurons as endogenous sources for RGC regeneration. Approaches could be potentially enhanced by modulation of signaling pathways that have already been shown to control the proliferation and neurogenic potential of Müller glia, such as Notch, JAK/STAT, HIPPO/YAP, EGF, WNT, and TGFß (Wan et al., 2012; Ueki and Reh, 2013; Yao et al., 2016; Hamon et al., 2019; Rueda et al., 2019; Sharma et al., 2020). The combined use of signaling modulators with neurogenic and/or RGC-specific transcription factors together with epigenetic remodeling may offer the optimal recipe.

Based on previous studies designed to regenerate optic pathways it is also clear that there are relevant aspects during optic nerve regeneration that apply to axon growth of transplanted or regenerated RGCs in regenerative strategies, such as: enhancing the intrinsic axon growth capacity of RGCs, overcoming the potential growth-inhibitory environment of the optic nerve in disease, and optimizing the signals responsible for reinnervation of the relevant targets. Recently it was shown that transplanted RGCs are able to integrate into the adult mouse retina and project axons to the superior colliculus and lateral geniculate nucleus (Venugopalan et al., 2016). In addition, these cells were responsive to light, with electrophysiological properties similar to endogenous RGCs (Venugopalan et al., 2016). This is a strong demonstration that the mature mammalian retina is not refractory to RGC integration. An important follow up is to investigate RGC integration and visual function recovery in disease context.

On the other hand, for the design of any regenerative approach, relevant technical challenges must be overcome, which include adequate lineage tracing strategies to guarantee the origin of the new neurons, either transplanted or endogenously generated, as well as the verification of a possible interference of direct protein transfer between donor and host cells in data interpretation, as recently debated (Pearson et al., 2016; Decembrini et al., 2017; Boudreau-Pinsonneault and Cayouette, 2018; Nickerson et al., 2018).

In the end, it is essential to define what specific properties replaced or regenerated RGCs must possess to effectively function as retinal projection neurons. We propose here that these essential properties are: transcriptional identity, integration and synaptic connectivity in the retina, response to light, and axon projection and targeting to proper brain areas (Figure 1).

Finally, the relevance of these studies for RGC replacement in humans is yet to be determined, and preclinical testing of promising strategies to revert vision loss will require the definition of the minimal number of regenerated or transplanted RGCs necessary to obtain useful visual recovery, and of how the long-term survival of integrated RGCs will be attained. Preclinical studies in non-human primate will likely be an important intermediate step to ensure success of any regenerative strategy. Despite the many barriers that remain, the rapid advances in our understanding of RGC development paves a path toward the ultimate goal of applying that knowledge to promote RGC replacement and vision restoration.

## Author Contributions

AB, VO-V, MV, and MS wrote and edited the manuscript. All authors contributed to the article and approved the submitted version.

## Conflict of Interest

The authors declare that the research was conducted in the absence of any commercial or financial relationships that could be construed as a potential conflict of interest.
